# Linfocitosis monoclonal de células B en familiares de pacientes colombianos con síndromes linfoproliferativos crónicos B

**DOI:** 10.7705/biomedica.7099

**Published:** 2023-12-29

**Authors:** Mike Celis, Yohanna Navarro, Norma Serrano, Daniel Martínez, Wendy Nieto

**Affiliations:** 1 Doctorado en Ciencias Biomédicas, Facultad de Salud, Universidad del Valle, Cali, Colombia Universidad del Valle Universidad del Valle Cali Cali; 2 Instituto de Investigación Masira, Facultad de Ciencias Médicas y de la Salud, Universidad de Santander, Bucaramanga, Colombia Universidad de Santander Universidad de Santander Bucaramanga Bucaramanga; 3 Grupo de Investigación Biomédica Traslacional, Hospital Internacional de Colombia, Floridablanca, Colombia Hospital Internacional de Colombia Hospital Internacional de Colombia Floridablanca Floridablanca

**Keywords:** linfocitosis, leucemia linfocítica crónica de células b, linfoma no Hodgkin, citometría de flujo, estudios de seguimiento, pruebas serológicas, Lymphocytosis, leukemia, lymphocytic, chronic, b-cell, lymphoma, nonHodgkin, flow cytometry, follow-up studies, serologic tests

## Abstract

**Introducción.:**

La linfocitosis monoclonal de células B, generalmente, precede la leucemia linfocítica crónica y afecta alrededor del 12 % de la población adulta sana. Esta frecuencia se incrementa en familiares de pacientes con síndromes linfoproliferativos crónicos de células B.

**Objetivo.:**

Determinar la frecuencia de linfocitosis monoclonal B en familiares de pacientes con síndromes linfoproliferativos crónicos B, sus características inmunofenotípicas y citogenéticas, posible relación con agentes infecciosos, y seguimiento a corto plazo de población colombiana.

**Materiales y métodos.:**

Se estudiaron 50 adultos sanos con antecedentes familiares de síndromes linfoproliferativos crónicos de célula B, empleando citometría de flujo multiparamétrica, pruebas citogenéticas y serológicas, encuesta de hábitos de vida y seguimiento a dos años.

**Resultados.:**

La frecuencia encontrada de linfocitosis monoclonal B fue del 8 %, con predominio del sexo femenino y edad avanzada, incrementándose al 12,5 % en individuos con antecedentes familiares de leucemia linfocítica crónica. Tres de cuatro individuos presentaron inmunofenotipo de tipo leucemia linfocítica crónica, todas con bajo recuento. A su vez, en estos individuos se observa de manera significativa un mayor número de células/ μl en subpoblaciones linfocitarias T, junto con mayor predisposición a la enfermedad. Las poblaciones clonales descritas aumentan a lo largo del tiempo de manera no significativa.

**Conclusiones.:**

La frecuencia y comportamiento de la linfocitosis monoclonal de célula B en pacientes con antecedentes familiares de síndromes linfoproliferativos crónicos B es similar a lo encontrado en estudios relacionados, lo que sugiere que no existe afectación de genes de mayor relevancia que puedan desencadenar una proliferación clonal descontrolada, pero que generan desregulación inmunológica que podría indicar un mayor riesgo de infección grave en estos individuos.

## Abstract (en)

**Introduction.:**

Monoclonal B-cell lymphocytosis generally precedes chronic lymphocytic leukemia, affecting about 12% of the healthy adult population. This frequency increases in relatives of patients with chronic B-cell lymphoproliferative disorders.

**Objective.:**

To determine the frequency of monoclonal B-cell lymphocytosis in relatives of patients with chronic B-cell lymphoproliferative disorders, their immunophenotypic/ cytogenetic characteristics, a possible relationship with infectious agents, and short-term follow-up in the Colombian population.

**Materials and methods.:**

Fifty healthy adults with a family history of chronic B-cell lymphoproliferative disorders were studied using multiparametric flow cytometry, cytogenetic/serological testing, lifestyle survey, and 2-year follow-up.

**Results.:**

The frequency of monoclonal B-cell lymphocytosis found was 8%, with a predominance of female gender and advanced age, increasing to 12.5% for individuals with a family history of chronic lymphocytic leukemia. Three out of four individuals presented chronic lymphocytic leukemia-type immunophenotype, all with low counts. In turn, a significantly higher number of cells/μΙ is observed in these individuals in T lymphocyte subpopulations, together with a greater predisposition to the disease. The described clonal populations increase over time in a non-significant manner.

**Conclusions.:**

The frequency and behavior of monoclonal B-cell lymphocytosis in patients with family history of chronic B-cell lymphoproliferative disorders are like those found in related studies, which suggests that there is no involvement of more relevant genes that can trigger uncontrolled clonal proliferation, but that generates immunological deregulation that could justify a greater risk of serious infection in these individuals.

Los síndromes linfoproliferativos crónicos de célula B, corresponden a un grupo de trastornos clonales de los linfocitos B, los cuales incluyen enfermedades como la leucemia linfocítica crónica, la leucemia prolinfocítica B, tricoleucemia, el linfoma de la zona marginal, el linfoma folicular, el linfoma de células del manto, la macroglobulinemia de Waldenstrom-linfoma linfoplasmocítico). Todas ellas son parcialmente similares en morfología celular, inmunofenotipo y genética molecular, pero significativamente diferentes en su tratamiento y pronóstico ^1^.

En su revisión del 2016 sobre neoplasias linfoides, la Organización Mundial de la Salud (OMS) incluyó la linfocitosis monoclonal de células B en el grupo de síndromes linfoproliferativos, a pesar de no ser una entidad con manifestaciones clínicas. La definió como la presencia de poblaciones monoclonales de linfocitos B de hasta 5 x 10^9^/ L en sangre periférica, cuyo fenotipo puede ser el de leucemia linfocítica crónica típica (LLC típica), de leucemia linfocítica crónica atípica (LLC atípica), o sin leucemia linfocítica crónica (no-LLC) ^2^.

La linfocitosis monoclonal de células B se encuentra hasta en el 12 % de la población adulta sana ^2^, con una media del 5 % ^3^. Esta frecuencia depende de la sensibilidad de la técnica empleada y aumenta considerablemente en familiares de pacientes con LLC-LLCP (LLCP: linfoma linfocitico de célula pequeña) hasta en el 18 % ^4^. Las poblaciones clonales B se mantienen en los individuos a lo largo del tiempo en el 90 % de los casos y la tasa de progresión de una linfocitosis monoclonal de células B de alto recuento a un síndrome linfoproliferativo crónico de células B clínicamente manifiesto, oscila entre el 1 y el 4 % anual (2,3); además, se sabe que esta linfocitosis parece comprometer el sistema inmunológico en estos individuos, lo cual genera un mayor riesgo de padecer complicaciones infecciosas graves ^5^.

Actualmente, en Colombia, en el único estudio en que se evaluó la linfocitosis monoclonal B en familiares con LLC, se encontró una frecuencia del 2 % ^6^, porcentaje muy bajo al comparar con otros estudios similares realizados hasta la fecha a nivel mundial ^4^^,^^7^.

Por tal motivo, en el presente trabajo se decidió cuantificar y caracterizar exhaustivamente la presencia de dichas poblaciones clonales, empleando diversas técnicas de análisis con la finalidad de detectar alteraciones numéricas, fenotípicas, séricas y moleculares; y, asimismo, relacionarlas con diferentes hábitos de vida que nos permitieran intuir su presencia o una probable evolución a corto plazo.

## Materiales y métodos

### Población y muestra

Se estudiaron 50 familiares en primer grado de consanguinidad de pacientes con síndromes linfoproliferativos crónicos de célula B mayores de 18 años y sin enfermedad hematológica, los cuales fueron reclutados mediante información suministrada en el Servicio de Medicina Nuclear y Oncología del Hospital Internacional de Colombia.

La participación en el estudio fue voluntaria y en ningún caso el participante fue identificado personalmente, manteniéndose el derecho a la privacidad y protección de su información personal según lo establecido por la ley colombiana estatutaria 1581 (*Habeas Data*) de 2012. Todos los individuos firmaron un consentimiento informado avalado por el comité institucional de ética en investigaciones y cada participante diligenció un cuestionario con información personal, familiar y de costumbres de rutina general, el cual se validó por juicio de expertos según suficiencia, claridad, coherencia y relevancia de las preguntas, con la finalidad de determinar factores que pudieran estar relacionados con la presencia de clones de linfocitosis monoclonal de células B. Se excluyeron del estudio las mujeres embarazadas.

En todos los casos, se obtuvo una muestra de 20 ml de sangre periférica anticoagulada con EDTA, con la cual se practicó un hemograma, se hizo el tamizaje para identificar linfocitosis monoclonal de células B por citometría de flujo, y se obtuvo una muestra de plasma para las pruebas serológicas. En los casos en los que se identificó una población de linfocitosis monoclonal B, se clasificó según el fenotipo; seguidamente, en algunos casos, se hizo la separación celular de la población en estudio para su análisis citogenético y posterior seguimiento a dos años de la identificación de la linfocitosis monoclonal B.

Todas las muestras se procesaron en un periodo no superior a 12 horas después de su recolección. La biometría hemática se llevó a cabo en el analizador hematológico automatizado (CELL-DIN Rubi™, Abbott Laboratories, Libertyville, IL), para conocer el recuento absoluto y porcentual de leucocitos.

### Estudio por inmunofenotipo

La identificación por inmunofenotipo de la linfocitosis monoclonal de células B se procesó según el protocolo descrito en Salamanca (España) ^8^, empleando la técnica de inmunofluorescencia directa con combinaciones múltiples de 12 anticuerpos en 8 canales de fluorescencia, según lo recomendado por el consorcio europeo *Euroflow* para el tamizaje de enfermedades linfoproliferativas ^9^.

Esto incluyó la siguiente combinación adaptada de anticuerpos: CD20- CD4/CD45/CD8-sIglambda/CD56-sIgkappa/CD5/CD19/CD3/CD38; este panel también permitió evaluar subpoblaciones de linfocitos T, células NK y linfoplasmocitos. Una vez realizadas las marcaciones de las muestras (9001.200 μl de sangre periférica), se obtuvo un mínimo de 5 x 10^6^ leucocitos totales por participante, empleando un citómetro de flujo BD FACSCanto II™ (Becton Dickinson).

Para el análisis de datos, se utilizó el *software* INFINICYTTM (Cytognos SL), contemplando un mínimo de 30 eventos de linfocitos B clonales para considerarla una población de linfocitosis monoclonal de células B. Los valores absolutos de linfocitos B se calcularon basados en el valor total de linfocitos en el hemograma.

En los participantes en quienes se detectó linfocitosis monoclonal B, la caracterización por inmunofenotipo se hizo mediante una ampliación de anticuerpos por citometría de flujo multiparamétrica de gran sensibilidad para determinar el tipo de linfocitosis monoclonal B (LLC típica, LLC atípica o noLLC), empleando las siguientes combinaciones de anticuerpos:

- CD20/CD45/CD200/CD23/CD19/CD10/CD5/CD38
- CD20/CD103/CD25/CD19/CD5/CD11c/CD49d/CD43

La compensación y calibración del citómetro se hicieron según las recomendaciones del consorcio *EuroFlow*^10^ y protocolos internos del laboratorio.

El seguimiento a los participantes con linfocitosis monoclonal de células B, se llevó a cabo en una media de 24 meses, empleando el panel de tamizaje descrito anteriormente. Los anticuerpos empleados en el estudio de inmunofenotipo fueron: CD20 BD Horizon™ V450 (BD, Cod. 642274), CD4 (SK3) V450 (BD, Cod. 651849), CD45 V500c (BD, Cod. 647449), CD8 FITC (BD, Cod. 347313), LAMBDA λ Light Chain FITC (BD, Cod. 346600), CD56 PE (BD, Cod. 347747), Kappa PE (BD, 346601), CD5 PerCP-Cy™5.5 (BD, Cod. 341089), CD19 PE-Cy™7 (BD, Cod. 341093), CD3 APC (BD, Cod. 340440), CD38 APC-H7 (BD, Cod. 656646), CD23 FITC (BD, Cod. 656148), CD200 APC (BD, Cod. 655406), CD10 PE (BD, Cod. 658366), CD103 FITC (BD, Cod. 340945), CD11c APC (BD, Cod. 658330), CD49d APC-H7 (BD, Cod. 658332), y CD43 APC-H7 (BD, Cod. 655407).

### Estudios serológicos

En todas las muestras de sangre periférica evaluadas, se recolectó un mínimo de 2 ml de plasma, el cual se almacenó a -80 °C para, posteriormente, realizar los estudios inmunológicos para los siguientes agentes infecciosos: Helicobacter pylori (IgG), virus del herpes simple (HSV) de tipo 1 y 2 (IgG), virus de la varicela zóster (HHV) (IgG), *Toxoplasma gondii* (IgG-IgM), virus de Epstein-Barr (EBV) (IgG-IgM), citomegalovirus (CMV) (IgG-IgM), virus de la hepatitis A (HAV) (IgG), virus de la hepatitis B (HBV) (HBsAg), virus de la hepatitis C (HCV) (IgG), y virus de la inmunodeficiencia humana (HIV) de tipo I y II (Ag-Ab).

Estas determinaciones se hicieron utilizando kits disponibles comercialmente para estudios inmunológicos, empleando los equipos Architect ci4100™ (Abbott) y el lector de microplaca iMark™ (BioRad), y siguiendo estrictamente las recomendaciones de los fabricantes.

Los kits empleados fueron: Architect Anti-HCV (Abbott, Ref. 10112339), Architect EBV VCA IgG (Abbott, Ref. 10171825), Architect EBV VCA IgM (Abbott, Ref. 10171826), Architect CMV IgM (Abbott, Ref. 10112360), Architect CMV IgG (Abbott, Ref. 10113034), Architect HAVAB IgG (Abbott, Ref.10136085), Architect HIV Ag/Ab (Abbott, Ref. 10142084), Architect Toxoplasma IgG (Abbott, Ref. 10136115), Architect Toxoplasma IgM (Abbott, Ref. 10136116), Architect HBSAG QUAL II (Abbott, Ref. 10144079), Herpes simple 1 ELISA IGG/IGM (Vircell, Ref. 988G/M1012), Herpes simple 2 ELISA IGG/IGM (Vircell, Ref. 988G/M1013), Varicela-zoster ELISA IGG/ IGM (VIRCELL, Ref. 988G/M1002) y Helicobacter pilory test (ABON, Ref. 105IPH302).

### Separación celular y caracterización citogenética

La purificación de estas poblaciones se realizó en el 50 % de los individuos con linfocitosis monoclonal de células B mediante la técnica de separación por citometría de flujo multiparamétrica con una pureza de separación de alrededor del 98 %, en el equipo BD FACSAria™ III (Becton Dickinson) del Instituto Nacional de Cancerología.

Los anticuerpos monoclonales empleados para la separación se seleccionaron teniendo en cuenta el inmunofenotipo de la población con dicha linfocitosis, encontrado en la muestra (LLC, LLC atípico, no-LLC). En todos los casos, se recogió la fracción B monoclonal y, como control, una fracción de células T de sangre periférica del individuo. La compensación y calibración del citómetro, y la metodología de separación, se llevaron a cabo según las especificaciones o recomendaciones del fabricante y siguiendo los protocolos internos del laboratorio.

Apenas se separaron las células, se sumergieron en solución de Carnoy y se llevaron al servicio de citogenética del Instituto Nacional de Cancerología. Allí se concentraron mediante centrifugación (Cytospin™) para, posteriormente, fijarlas directamente en portaobjetos y practicar la hibridación fluorescente *in situ* (*Fluorescence in situ Hybridization*, FISH) en interfase.

Las sondas empleadas fueron la Vysis LSI IGH Dual Color Break Apart (Abbott, Ref. 08L63-020) y la Vysis LSI MLL Dual Color Break Apart (Abbott, Ref. 08L57-020). En cada fracción separada se contaron los puntos de hibridación de todos los núcleos obtenidos por prueba citogenética.

### Análisis estadístico

Para las variables continuas, se calcularon promedios, medianas, y valores máximos y mínimos, mientras que para las variables discretas se utilizaron tablas de frecuencias absolutas y relativas. Se establecieron comparaciones bivariadas entre la presencia o la ausencia de linfocitosis monoclonal de células B y las variables continuas, utilizando la prueba U de Mann-Whitney para muestras independientes.

Para establecer la asociación entre dicha linfocitosis (como valor absoluto o porcentual) y la edad de los participantes, se utilizó un modelo de regresión lineal simple. Para establecer la relación de la presencia o ausencia de la linfocitosis con las variables discretas de las pruebas serológicas y las variables establecidas a partir de la encuesta, se utilizó la prueba exacta de Fisher.

Además, se estableció la relación entre el número absoluto de linfocitos monoclonales B en el momento de la obtención de los datos en la línea base, y el encontrado en la evaluación practicada en el seguimiento a los dos años, en los individuos con reporte inicial de presencia de linfocitosis monoclonal B, utilizando la prueba de rangos con signo para muestras pareadas.

El análisis estadístico de los datos se hizo con el paquete estadístico Stata™ (Stata Corp. LLC, Texas, USA) y, como parámetro de significancia, se utilizó el valor de p menor de 0,05.

### Consideraciones éticas

El presente estudio cumplió con las disposiciones de la Declaración de Helsinki, y las normas científicas, técnicas y administrativas para la investigación en salud, y fue aprobado por el Comité de Ética en Investigación de la Fundación Cardiovascular de Colombia.

## Resultados

En cuatro de los 50 individuos evaluados en el estudio, se identificaron poblaciones de linfocitos monoclonales B, lo que correspondió a una frecuencia de presentación del 8 %; la frecuencia fue del 10 % (3/30) en las mujeres y del 5 % (1/20) en hombres. Se observó que dicha frecuencia aumentaba con la edad: fue del 4,3 % entre los 18 y los 40 años, del 9,5 % entre los 41 y los 60 años, y del 16,7 % en mayores de 60 años. No se detectaron diferencias estadísticamente significativas entre el sexo, la edad y la presencia de linfocitosis monoclonal B (cuadro 1).

**Cuadro 1.** Frecuencia y número de linfocitos monoclonales B relacionados con la edad y el sexo en familiares de pacientes con síndromes proliferativos de células B

	Sexo			P	Rango de edad (años)			P
	Total	Femenino	Masculino		18-40	41-60	>60	
Participantes	50	30	20		23	21	6	
Participantes con LMB [n (%)]	4 (8)	3 (10)	1 (5)	0,527	1(4,3)	2 (9,5)	1(16,7)	0,142
Media (%) y rango de LMB/linfocitos	0,13	0,06	0,33	0,179	0,33	0,08	0,01	0,148
Media y rango LMB/μl	(0,01-0,33)	(0,01-0,11)				(0,05-0,11)		
	3,9	2,1	9,3	0,179	9,3	2,9	0,33	0,162
	(0,33-9,3)	(0,33-4,47)				(1,4-4,5)		

LMB: linfocitosis monoclonal de células B

Diferencia estadísticamente significativa: p<0,05)

Asimismo, de los cuatro individuos con dicha linfocitosis, en tres fue de tipo LLC y en uno fue de tipo no-LLC. En todos, la linfocitosis mostró un recuento escaso y solo en uno fue biclonal. La media encontrada de células B clónales fue de 3,9/μΙ y la mediana fue de 2,9/μΙ (rango: 0,3-9,3). En concordancia con estudios similares, se identificó una mayor frecuencia en individuos con antecedentes familiares de linfocitosis monoclonal B (12,5 %) que en aquellos casos con familiares con otros síndromes linfoproliferativos de células B (7,1 %); el individuo con la linfocitosis de tipo no-LLC presentó el inmunofenotipo CD5-/CD19+/CD38-/CD20+higth/CD23-/CD49d+/KAPPA+.

En relación con las variables del hemograma, hubo diferencias estadísticamente significativas en el recuento absoluto de basófilos/μΙ, entre los participantes con la linfocitosis y aquellos sin la enfermedad (p=0,015; IC_95%_: 26,08 a 75,37); esta población de basófilos fue, en promedio, más numerosa en los participantes con linfocitosis monoclonal de células B (84/ μl). De igual forma, se observaron diferencias significativas y un mayor promedio de células/μl en la población de linfocitos T CD4 (p=0,0136; IC_95_%: 142,28-692,78), CD8 (p=0,0415; IC_95%_: 11,73-468,26) y CD4/CD8 positivos (p=0,0411; IC_95%_: 1,52-6,10) (cuadro 2).

**Cuadro 2.** Relación de la linfocitosis monoclonal B con diferentes poblaciones celulares presentes en sangre periférica

	Total (n=50)	LMB (n=4)	No-LMB (n=46)	P
Leucocitos	7.581,7 (4.600-16.200)	10.580 (7.180-16.200)	7.366 (4.600-13.300)	0,062
Linfocitos	2.611 (1.370-4.330)	3.230 (2.760-4.060)	2.557 (1.370-4.330)	0,068
Monocitos	560 (278-1.180)	642 (454-910)	553 (278-1.180)	0,39
Neutrófilos	4.093 (1.880-11.200)	5.983 (3.140-1.200)	3.851 (1.880-8.680)	0,198
Eosinófilos	269 (0-1.333)	167 (0-464)	278 (36-1.333)	0,174
Basófilos	37 (0-175)	84 (42-175)	33 (0-83)	0,015
Plaquetas	283.680 (16.100-409.000)	290.000 (180.000-409.000)	283.130 (161.000-364.000)	0,858
Linfocitos B	276,7 (87-743)	279,1 (212-418)	276,5 (87-743)	0,591
Linfocitos T	1484 (548-2.568)	2083 (1.604-2.301)	1432 (548-2.568)	0,591
CD4	843 (322-1.567)	1223 (849-1.567)	805 (322-1.516)	0,013
CD8	556 (146-1.126)	777 (611-956)	524 (146-1.126)	0,041
CD4+/CD8+	2,7 (0-11,5)	6,3 (1,4-11,5)	2,7 (0-9,2)	0,041
Células NK	340 (102-773)	336 (149-462)	340 (102-773)	0,774
Dendríticas plasmocitoides	22 (0-61)	34 (0-61)	21 (0-51)	0,333
Dendríticas monocitoides	67 (0-163)	36 (0-59)	69,7 (0-163)	0,111
Linfoplasmocitos	10 (0-138)	3 (0-24)	10 (0,9-138)	0,816

LMB: linfocitosis monoclonal de células B; n: número de individuos; NK: *natural killer*

Se representa el promedio (mínimo-máximo), todos los valores se expresan en μl.

Diferencia estadísticamente significativa (p<0,05).

Una vez analizadas las variables descritas en la encuesta de los 50 participantes con antecedentes familiares de síndrome linfoproliferativo crónico B, se hallaron diferencias estadísticamente significativas entre los individuos con y aquellos sin poblaciones de linfocitos monoclonales B para las variables de reducción de labores por enfermedad reciente (p=0,016; IC_95%_: -0,98 a -0,25), donde la población con linfocitos monoclonales B tiende a presentarlas más a menudo (75 %) en comparación con los individuos sin esta condición (13 %). Asimismo, se encontraron diferencias significativas en relación con la frecuencia de actividad física (p=0,051; IC_95%_: -0,97 a 1,23) y se observó mayor sedentarismo en la población con linfocitosis monoclonal de células B (cuadro 3).

**Cuadro 3.** Características epidemiológicas, sociodemográficas y de hábitos de vida, en pacientes con linfocitosis monoclonal B, en comparación con aquellos sin esta enfermedad

		LMB	No-LMB	P
Total		4	46	
Estado de salud general (%)				
	Excelente	0,0	17,4	1
	Muy buena	0,0	10,9	
	Buena	75,0	50,0	
	Regular	25,0	21,7	
	Mala	0,0	0,0	
Limitación con el esfuerzo intenso (%)				
	Sí, me limita mucho.	0,0	10,9	0,12
	Sí, me limita un poco.	75,0	26,1	
	No, no me limita nada.	25,0	63,0	
Limitación con el esfuerzo moderado (%)				
	Sí, me limita mucho.	0,0	6,5	0,603
	Sí, me limita un poco.	25,0	13,0	
	No, no me limita nada.	75,0	80,5	
Limitación al agacharse o arrodillarse (%)				
	Sí, me limita mucho.	0,0	6,5	1
	Sí, me limita un poco.	0,0	17,4	
	No, no me limita nada.	100,0	76,1	
Reducción de labores por enfermedad reciente (%)				0,016
	Sí	75,0	13,0	
	No	25,0	87,0	
Reducción de labores por afección emocional o psicológica reciente (%)				
	Sí	50,0	8,7	0,066
	No	50,0	91,3	
Manifiesta algún nivel de dolor (%)				
	Nada	25,00	60,9	0,32
	Un poco	25,00	10,9	
	Regular	50,00	17,4	
	Bastante	0,00	6,5	
	Mucho	0,00	4,3	
Sensación de vitalidad (%)				
	Siempre	25,0	30,4	0,118
	Casi siempre	25,0	28,3	
	Muchas veces	0,0	17,4	
	Algunas veces	25,0	23,9	
	Solo alguna vez	25,0	0,0	
	Nunca	0,0	0,0	
Sensación de calma (%)				
	Siempre	0,0	41,3	0,179
	Casi siempre	25,0	26,1	
	Muchas veces	25,0	13,0	
	Algunas veces	50,0	19,6	
	Solo alguna vez	0,0	0,0	
	Nunca	0,0	0,0	
Sensación de agotamiento (%)				
	Siempre	0,0	0,0	0,458
	Casi siempre	0,0	0,0	
	Muchas veces	25,0	6,5	
	Algunas veces	50,0	45,7	
	Solo alguna vez	0,0	21,7	
	Nunca	25,0	26,1	
Afección de la vida social por problemas (%)				
	Siempre	0,0	2,2	0,632
	Casi siempre	25,0	6,5	
	Muchas veces	0,0	0,0	
	Algunas veces	25,0	28,3	
	Solo alguna vez	0,0	13,0	
	Nunca	50,0	50,0	
Consumo de cigarrillo (%)				
	Sí	0,0	4,3	0,286
	No	50,0	80,5	
	Exfumador	50,0	15,2	
Consumo de alcohol (%)				
	Diario	0,0	2,1	1
	Esporádicamente	25,0	28,3	
	Fines de semana	0,0	8,7	
	Nunca	75,0	60,9	
Consumo frecuente de medicamentos (%)				
	Sí	75,0	50,0	0,611
	No	25,0	50,0	
Actividad física (%)				
	De pie, sin grandes desplazamientos	75,0	19,6	0,051
	Caminando, llevando algún peso, desplazamientos frecuentes	0,0	52,2	
	Trabajo pesado, gran esfuerzo físico	25,0	26,1	
	De pie, sin grandes desplazamientos	0,0	2,1	
Rutina de ejercicio en tiempo libre (%)				
	No hago ejercicio.	50,0	50,0	0,671
	Alguna actividad física o deportiva	25,0	21,7	
	Actividad física regular	0,0	19,6	
	Entrenamiento varias veces a la semana	25,0	8,7	
Enfermedad relevante (%)
	Sí	100,0	47,8	0,111
	No	0,0	52,2	
Antecedentes familiares de enfermedad relevante (%)
	Sí	100,0	100,0	NA
	No	0,0	0,0	
Padecimiento de enfermedades infecciosas (%)				
	Sí	0,0	41,3	0,284
	No	100,0	58,7	
Estado laboral (%)			
	Trabajador activo	75,0	47,8	0,119
	Desempleado	0,0	13,0	
	Jubilado	25,0	2,2	
	Sus labores	0,0	37,0	
Exposición a agente extraño (%)				
	Sí	25,0	28,3	0,69
	No	75,0	71,7	
Nivel de estrés (%)				
	No manifiesta	50,0	28,3	0,638
	Un poco	0,0	13,0	
	Regular	0,0	30,4	
	Bastante	50,0	28,3	
	Mucho	0,0	0,0	
Satisfacción laboral (%)				
	Nada	0,0	0,0	0,545
	Un poco	0,0	6,5	
	Regular	25,0	8,7	
	Bastante	50,0	60,9	
	Mucho	25,0	23,9	

NA: no aplica; LMB: linfocitosis monoclonal de células B Diferencia estadísticamente significativa: p<0,05

**Figura 1.**
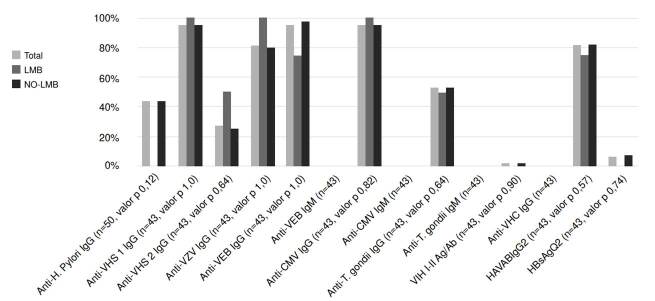
Frecuencia de seropositividad en todos los pacientes con linfocitosis monoclonal de células B y en aquellos sin esta enfermedad

CMV: citomegalovirus; HAVAB: anticuerpos para virus de la hepatitis A; HBsAgQ2: antígeno de superficie para hepatitis B; EBV: virus de Epstein-Barr; HIV: virus de la inmunodeficiencia humana; HCV: virus de la hepatitis C; HSV: virus del herpes simple; HHV-3: virus de la varicela y el herpes zóster

Los resultados obtenidos de las pruebas serológicas no fueron relevantes, ya que no se observaron diferencias significativas al comparar los individuos con presencia y aquellos con ausencia de poblaciones con linfocitosis monoclonal de células B, y los resultados de seropositividad obtenidos para los diferentes agentes infecciosos estudiados (figura 1).

Diferencia estadísticamente significativa (p<0,05).

Las pruebas FISH realizadas para identificar alteraciones genéticas en poblaciones de linfocitosis monoclonal de células B separadas por citometría de flujo, determinaron anomalías en dos de los cuatro individuos; se encontraron alteraciones cromosómicas para el gen *IGH* en el 50 % de las células analizadas del individuo con inmunofenotipo no-LLC y, reordenamientos en *MLL* para un 27,3 % de los núcleos evaluados en el individuo con fenotipo de tipo LLC. Sin embargo, en ninguno de los dos adultos estudiados se logró analizar un mínimo de 50 núcleos, a consecuencia de la baja proporción de linfocitos monoclonales B en ellos, lo que también limitó la realización de pruebas en los otros dos individuos con poblaciones clonales B.

La media de seguimiento de los individuos con poblaciones con linfocitosis monoclonal de células B fue de 26 meses (rango: 23-28 meses); ninguno de los casos identificados progresó a LLC o a otro síndrome linfoproliferativo de células B.

De los cuatro adultos con poblaciones clonales, el número absoluto de las células B anormales aumentó en uno, disminuyó en dos y se mantuvo igual en el otro; sin embargo, no se observan diferencias estadísticamente significativas entre el valor absoluto de linfocitosis monoclonal de células B hallado inicialmente y el encontrado en el seguimiento, como se ha descrito de forma similar en estudios relacionados hasta la fecha (cuadro 4).

**Cuadro 4.** Frecuencia y número de linfocitosis monoclonal de células B al inicio y después del seguimiento a los dos años en los cuatro casos

	LMB inicial		LMB seguimiento	P
%	Número absoluto de linfocitos B clonales/μl	%	Número absoluto de linfocitos B clonales/μl	
0,01	0,3	0,01	0,3	1
0,33	9,3	0,9	25,4	
0,05	1,4	0,01	0,2	
0,11	4,5	0,07	3,3	

LMB: linfocitosis monoclonal de células B

%: porcentaje de linfocitos B clonales

Diferencia estadísticamente significativa (p<0,05).

## Discusión

Diversas publicaciones realizadas en individuos con antecedentes familiares de LLC revelaron una frecuencia de linfocitosis monoclonal de células B similar a la encontrada en el presente estudio, la cual se ubica entre el 13 y el 18 % ^4^^,^^7^, y muy superior a la identificada en el único estudio reportado en Colombia, tal vez a consecuencia de la cantidad de células analizadas para cada experimento, ya que el volumen de muestra evaluado en este trabajo fue de dos a tres veces superior.

Sin embargo, esta frecuencia disminuyó frente a individuos con antecedentes familiares de síndrome linfoproliferativo de células B diferentes a la LLC. Al igual que en otros trabajos publicados, la linfocitosis monoclonal B identificada con mayor frecuencia fue la LLC típica de bajo recuento y con predominio en el sexo femenino ^6^^,^^7^.

Otros autores describen diferentes alteraciones en poblaciones de linfocitos T, de células NK o en ambas, y reportan un incremento en individuos con linfocitosis monoclonal B ^11^^,^^12^; esto se evidenció en el presente estudio de manera significativa en subpoblaciones de linfocitos T, lo que podría indicar una probable desregulación inmunológica presente en estos individuos ^12^. Este incremento es atribuido a una posible reacción celular inmunitaria frente a estas células B clonales.

Es de resaltar que en los estudios realizados hasta la fecha sobre linfocitosis monoclonal B, no se ha descrito incremento en la proporción de basófilos. Sin embargo, se sabe que estas células también expresan el marcador CD22 de linaje B y, por ende, se deduce que estos pueden regular algunas de las señales de activación estimuladas por el complejo antígeno-IgE por medio de FcεR1, algo similar a la regulación de señales de activación en linfocitos B estimulados por antígeno mediante el complejo receptor de células B/CD79 ^13^^,^^14^.

Entre los principales hallazgos obtenidos de la encuesta sobre hábitos de vida, se apreció una mayor frecuencia de factores que suelen estar relacionados con diferentes tipos de cáncer, como el mayor consumo de cigarrillo en la población con linfocitosis monoclonal de células B; aun así, no se observaron diferencias estadísticamente significativas, lo que posiblemente se deba a que, en este tipo de personas, la presencia de clones B pudo originarse por factores genéticos hereditarios ^15^. Por otra parte, se identificaron significancias relacionadas con el menor grado de actividad física y una mayor reducción de actividades por enfermedad reciente, en las personas con linfocitosis monoclonal de células B en relación con aquellas sin linfocitosis monoclonal de células B.

Actualmente, no hay estudios sobre esta linfocitosis que la vinculen con sedentarismo; sin embargo, el estilo de vida sedentario, la dieta poco saludable y el tabaquismo son factores de riesgo conocidos para diversas enfermedades, incluidos los trastornos hematológicos y el cáncer ^16^.También, existe evidencia que sugiere que algunos de los déficits inmunológicos observados en la linfocitosis monoclonal de células B de alto recuento están presentes en aquella con escaso recuento, aunque en menor grado, lo que podría soportar la mayor reducción de actividades por enfermedad en los individuos con linfocitosis monoclonal de células B en el presente trabajo ^5^^,^^17^. Esto también podría estar apoyado por la aparente desregulación celular del sistema inmunológico observada en estos individuos.

Con respecto a los análisis serológicos, no se encontraron diferencias significativas con ninguno de los agentes patógenos infecciosos evaluados, lo que nos llevó a presumir que, en estos individuos, no existe ninguna asociación entre la linfocitosis monoclonal de células B encontrada y la infección con alguno de los agentes estudiados. Por ende, su presencia se atribuiría más bien a un proceso normal del sistema inmunitario, el de inmunosenescencia, intensificado por factores hereditarios que aumentan su frecuencia en los individuos de mayor edad y las personas con antecedentes familiares de LLC ^6^^,^^15^.

Asimismo, el hecho de que se observaran alteraciones citogenéticas en los dos individuos con linfocitosis monoclonal de células B estudiados, sustentó los hallazgos de otros investigadores que demuestran que la acumulación del daño en el ADN y la alteración en sus mecanismos de reparación, son características críticas de la inestabilidad genética, por lo cual se presume su implicación en la patogenia de la linfocitosis monoclonal de células B y otros síndromes linfoproliferativos de células B ^18^. Los reordenamientos del gen MLL generan una proliferación selectiva y una ventaja de supervivencia en las células leucémicas; no obstante, es insuficiente por sí solo para inducir la leucemogénesis y, por lo tanto, son necesarios estímulos oncogénicos adicionales ^19^.

Por otra parte, las translocaciones que involucran el gen *IGH* se presentan, aproximadamente, en el 50 % de los síndromes linfoproliferativos crónicos B y ocurren temprano en el proceso de transformación clonal; aun así, tampoco parecen afectar el riesgo de progresión de la linfocitosis a su fase clínica. Sin embargo, la adquisición de anomalías clonales, junto con el aumento del recuento de células B, son los principales factores determinantes para el progreso de la enfermedad ^20^^,^^21^.

A pesar del aumento en la mediana del tamaño del clon B observado después del seguimiento, no hubo diferencias significativas. Por lo tanto, se podría concluir que, muy posiblemente, no se han afectado genes de mayor relevancia para desencadenar una proliferación clonal descontrolada en estos casos (por lo cual, probablemente, no sea una afección preleucémica); no obstante, como lo sugieren otros autores, el riesgo de padecer infecciones graves es más importante ^5^.

Finalmente, se recomienda hacer futuros estudios sobre el tema, en los que se involucren poblaciones más grandes de individuos con antecedentes familiares de síndrome linfoproliferativo de células B y que permitan corroborar algunos de los hallazgos descritos por primera vez en este trabajo. Asimismo, se sugiere el seguimiento a largo plazo, ya que los estudios existentes a la fecha incluyen solo adultos sanos y no a familiares de pacientes con diferentes síndromes linfoproliferativos de células B.
